# Membrane potential dynamics of C5a-stimulated neutrophil granulocytes

**DOI:** 10.1007/s00424-024-02947-8

**Published:** 2024-04-13

**Authors:** Stina Becker, Aljoscha Swoboda, Henrik Siemer, Sandra Schimmelpfennig, Sarah Sargin, Victor Shahin, Albrecht Schwab, Karolina Najder

**Affiliations:** 1 Institute of Physiology II, University Hospital Münster, Münster, Germany; 2 Department of Anesthesiology, Intensive Care and Pain Medicine, University Hospital Münster, Münster, Germany

**Keywords:** Neutrophils, Membrane potential dynamics, Chemotaxis, ROS

## Abstract

Neutrophil granulocytes play a crucial role in host defense against invading pathogens and in inflammatory diseases. The aim of this study was to elucidate membrane potential dynamics during the initial phase of neutrophil activation and its relation to migration and production of reactive oxygen species (ROS). We performed ROS production measurements of neutrophils from healthy C57BL/6J mice after TNFα-priming and/or C5a stimulation. The actin cytoskeleton was visualized with fluorescence microscopy. Furthermore, we combined migration assays and measurements of membrane potential dynamics after stimulating unprimed and/or TNFα-primed neutrophils with C5a. We show that C5a has a concentration-dependent effect on ROS production and chemokinetic migration. Chemokinetic migration and chemotaxis are impaired at C5a concentrations that induce ROS production. The actin cytoskeleton of unstimulated and of ROS-producing neutrophils is not distributed in a polarized way. Inhibition of the phagocytic NADPH oxidase NOX2 with diphenyleneiodonium (DPI) leads to a polarized distribution of the actin cytoskeleton and rescues chemokinetic migration of primed and C5a-stimulated neutrophils. Moreover, C5a evokes a pronounced depolarization of the cell membrane potential by 86.6 ± 4.2 mV starting from a resting membrane potential of -74.3 ± 0.7 mV. The C5a-induced depolarization occurs almost instantaneously (within less than one minute) in contrast to the more gradually developing depolarization induced by PMA (lag time of 3—4 min). This initial depolarization is accompanied by a decrease of the migration velocity. Collectively, our results show that stimulation with C5a evokes parallel changes in membrane potential dynamics, neutrophil ROS production and motility. Notably, the amplitude of membrane potential dynamics is comparable to that of excitable cells.

**Supplementary Information:**

The online version contains supplementary material available at 10.1007/s00424-024-02947-8.

## Introduction

Neutrophil granulocytes are major components of the innate immune response. Their ability to migrate toward a site of infection or injury determines how rapidly the danger will be recognized and fought. Moreover, well-balanced neutrophil killing mechanisms are pivotal for taming an infection and its successful resolution [24]. Extensive activation can cause severe tissue injury as in rheumatoid arthritis, whereas neutrophil defects result in life-threatening infections [44]. Therefore, neutrophil migration and other effector functions must be executed timely and sequentially.

Ligand binding to FcγRs and G-protein coupled receptors (GPCRs) as well as interaction with selectins and adhesion molecules on endothelial cells lead to electrogenic ion fluxes in neutrophils [21]. Most notably, an increase of the intracellular Ca^2+^ concentration ([Ca^2+^]_i_), elicited by store-operated Ca^2+^ entry (SOCE) triggers a cascade of signaling pathways modulating actin dynamics [45], reactive oxygen species (ROS) production [15], exocytosis and release of neutrophil extracellular traps (NETs) (reviewed in [17]). Activation of phagocytic NADPH oxidase (NOX2) results in electron transport out of the cell or into phagocytic vesicles and acidification of the cytosol [7]. Both [Ca^2+^]_i_ increase [16] and ROS production by NOX2 contribute to a massive depolarization of the neutrophil membrane. However, the consequences of membrane potential dynamics in neutrophils are not yet well understood.

Here we show that neutrophil migration and ROS production are rather exclusive and depend on the type and concentration of the trigger. Our analysis of neutrophil migration, actin polymerization and concurrent membrane depolarization shows that TNFα priming and/or high concentration of chemotactic stimulus dampen neutrophil migration but enhance ROS production. Conversely, neutrophil migration is preserved under these conditions when NOX2 is inhibited. Moreover, we tested whether membrane potential dynamics could be one of the determinants of neutrophil migration and ROS production and thereby modulate neutrophil behavior.

## Materials and methods

### Mice

We used 8–14 weeks old male C57BL/6 J (wild type, WT) mice in all experiments. Mice were euthanized by cervical dislocation. Experimental protocols were approved by the local committee for animal care with permit number: 84–02.05.50.15.010.

### Reagents

Reagents were purchased from SigmaAldrich, Steinheim, Germany if not indicated otherwise.

### Cell culture

Murine myelocytic leukemia (WEHI-3B)-conditioned medium was prepared as described earlier [28]. Shortly, WEHI-3B cells were cultivated in bicarbonate-buffered DMEM containing 4 mM L-glutamine, 10% FCS (ThermoFisher), 100 U/ml penicillin and 100 µg/ml streptomycin at 37 °C in a humidified atmosphere containing 5% CO_2_ until confluent. The cells were pelleted (200 g, 15 min) and the supernatant was collected and sterile filtered. Aliquots were frozen and stored for later use.

### Neutrophil isolation

We used non-pyrogenic labware for neutrophil handling throughout the study. Neutrophils were isolated as described previously [28]. Tibiae and femora were isolated and flushed with 10 ml of cold Ca^2+^/Mg^2+^-free HBSS (HBSS^−/−^) containing 25 mM HEPES and 10% FCS (washing buffer). The resulting pellet was resuspended in 1 ml of cold washing buffer and filtered through a 70 µm mesh into fresh washing buffer. After centrifugation (4 °C, 200 g, 10 min), the pellet was resuspended in 1 ml HBSS^−/−^ with 20 mM HEPES and added on top of a Histopaque 1077/Histopaque 1119 gradient and centrifuged (22 °C, 650 g, 30 min). The neutrophil-containing layer was resuspended twice in washing buffer and centrifuged (4 °C, 200 g, 10 min). Finally, the pellet was resuspended in 10 ml RPMI 1640 medium supplemented with 10% WEHI-3B-conditioned medium, 10% heat-inactivated FCS, 100 U/ml penicillin and 100 µg/ml streptomycin. The neutrophils were incubated at 37 °C in a humidified atmosphere of 5% CO_2_ overnight. The purity of the isolated neutrophils was evaluated based on the Ly6G positivity, which amounts to ~ 85% [34].

### Measurement of ROS production

Following overnight incubation, neutrophils (1–2 × 10^6^) were pelleted (4 °C, 200 g, 10 min) and suspended in 1 ml Ringer’s solution (containing 122.5 mM NaCl, 1.2 mM CaCl_2_, 0.8mM MgCl_2_, 5.4 mM KCl, 10 mM HEPES, 5.5 mM glucose, adjusted to pH 7.4 with 1N NaOH). ROS was quantified according to a modified method described earlier [26]. Neutrophils were incubated at 37°C with dihydrorhodamine-123 (DHR-123) and without or with TNFα (5; 10; 20 ng/ml) for 15 min prior to the stimulation with C5a or PMA. Membrane permeable DHR-123 once oxidized remains in the cytosol and produces a fluorescent signal when excited at 485 nm. Cells were then stimulated with 100 nM phorbol myristate acetate (PMA) or 60 nM C5a at 37°C for 15 min. The reaction was stopped by adding ice-cold Ringer’s solution. Neutrophils were pelleted (4 °C, 200 g, 5 min), resuspended in Ringer’s solution and then subjected to fluorescence analysis (Emission: 538 nm; Fluoroskan plate reader, Labsystems Diagnostics Oy, Helsinki).

### Neutrophil priming and stimulation

Following overnight incubation, cells were pelleted (25°C, 200 g, 10 min) and resuspended in 37°C warm Ringer’s solution. Suspended neutrophils were primed with TNFα (20 ng/ml = 1.16 nM) in the absence or presence of diphenyleneiodonium (DPI) (10 µM) for 10 min. Unprimed neutrophils served as control. Chemokinetic migration of neutrophils was induced with 6 nM or 60 nM C5a in the absence or presence of TNFα (20 ng/ml) and/or DPI (10 µM).

### Chemokinesis and chemotaxis experiments

For chemokinesis, neutrophils were seeded in fibronectin-coated (25 µg/ml) Ibidi Slides (µ-slide type I). Neutrophil chemotaxis was monitored in a confined 3D collagen-I-matrix (pH 7.4) containing 20.05 µl H_2_O, 5.46 µl 10 × HBSS^−/−^, 1.25 µl 1M HEPES, 0.64 µl 1M NaOH, 22.6 µl collagen I (9.29 mg/ml) and 50 µl of the cell suspension. This collagen-I-matrix containing the neutrophils was added to fibronectin-coated (25 µg/ml) Ibidi slides (µ-slide type I; Ibidi, Gräfelfing, Germany) [28].

After 10 min incubation neutrophil migration was recorded in heating chambers at 37 °C for 30 min. We used Zeiss Axiovert 40 C and Axiovert 25 microscopes and acquired the images with Bresser MikroCam SP 3.1 cameras. Images were taken every 5 s using MikroCamLab II software. Prior to chemokinesis experiments, all slides were washed with 300 µl Ringer with 5mM glucose and the respective compounds to remove non-adherent cells.

The migration assays were analysed by tracking the centers of 20 neutrophils for each experiment (N) with Amira Software (Thermo Fisher). Thus, migration is defined as the movement of the cell center per time unit. The velocity (v) was calculated by applying a three-point difference quotient [11]. Translocation (T) is defined as the net distance between the positions of the neutrophils at the start and the end of the experiment. The total path length (P) is the cumulative distance covered during the time of observation. The chemotaxis index (CI) is defined as the quotient of the net movement into the direction of the chemoattractant gradient and the total path length (P).

### Actin polymerization and neutrophil polarization

To assess the distribution of polymerized actin in unstimulated and in chemokinetically stimulated neutrophils we stained the actin cytoskeleton with TRITC-phalloidin. Following chemokinetic migration experiments, neutrophils were fixed by adding 4% PFA in PBS^+/+^ and incubated at RT for 1h. The cells were then washed with 1ml PBS^+/+^ and permeabilized with 0.1% Triton X-100 in PBS^+/+^ for 3 min. Unspecific binding sites were blocked with 10 % FCS for 30 min. Actin was stained by adding TRITC-labelled phalloidin (1:100, Thermo Fisher) in PBS^+/+^ with 10% FCS for 1h. Afterwards, cells were washed with PBS containing 1 µg/ml DAPI.

Actin distribution in neutrophils was analysed by means of fluorescence microscopy (Zeiss Observer.D1, 40x). Zeiss AxioCam MRm camera was controlled by Micro-Manager 2.0 gamma software. We acquired ten images for each experiment. The neutrophils were identified as Ly6G^+^ and based on the characteristic shape of their DAPI-stained nuclei. Distribution of polymerized actin was evaluated with ImageJ. We quantified the background-corrected mean fluorescence intensity (MFI) of ≧10 TRITC-phalloidin stained neutrophils in each image. Moreover, we determined the percentage of neutrophils with a polarized distribution of the actin cytoskeleton in these images.

### Membrane potential measurements using fluorescence imaging

We measured the membrane potential according to a modified protocol described earlier [23]. Following overnight incubation neutrophils were pelleted (25 °C, 200 g, 10 min) and resuspended in 1 ml 37 °C warm Ringer’s solution. After adding 2 µM DiBaC_4_(3) neutrophils were plated onto fibronectin-coated (25 µg/ml) Ibidi Slides (µ-slide type I) and incubated at 37 °C for 10 min. Then cells were washed with Ringer’s solution containing 2 µM DiBaC_4_(3) and 20 ng/ml TNFα when indicated and placed on the heated stage of an inverted fluorescence microscope (Zeiss Axiovert 200; 40 × objective) equipped with a pco.edge camera. DiBaC_4_(3) fluorescence was excited at 490 nm in 20 s intervals using a VisiChrome High Speed Polychromator System (Visitron Systems, Puchheim, Germany). Fluorescence emission was recorded at 520 nm. Data acquisition was controlled by VisiView software (Visitron Systems). Fluorescence intensity was measured over the whole cell area and was corrected for background fluorescence with ImageJ.

Measurements started with a ~ 3 min control period during which neutrophils were superfused with Ringer’s solution adjusted to contain exactly 140 mM NaCl and 2 µM DiBaC_4_(3) without or with TNFα (20 ng/ml). This was followed by 6 min of stimulation with 60 nM C5a or 100 nM PMA in the above-mentioned Ringer’s solution. At the end of each experiment, the measurements were calibrated by sequentially applying Ringer's solutions with 2 µg/ml gramicidin and 2 µM DiBaC_4_(3) that contained 2 mM, 35 mM or 140 mM NaCl; when necessary NaCl was isosmotically replaced by N-methyl-D-glucamin (NMDG)-chloride. DiBaC_4_(3) fluorescence of each neutrophil was calibrated separately with "its own" calibration parameters that were determined by linear regression. We only included those neutrophils in our analysis whose calibration coefficient of the regression was r^2^ ≥ 0.95. Published reports indicate that DiBaC_4_(3) can cover membrane potential values ranging from -180 mV to + 40 mV [25].

We used the Goldman-Hodgkin-Katz equation to calculate the absolute membrane potential values. We assumed an intracellular K^+^ concentration of 140 mM and intracellular Na^+^ concentration of 12 mM, referring to our recently published data [32].

Following the analysis of membrane potential dynamics of individual neutrophils, we also quantified migration of these neutrophils as described above. This allowed the precise temporal correlation of membrane potential dynamics with migratory behavior.

### Statistical analysis

All experiments were done in at least three biological replicates. All data is shown as mean values ± standard error of the mean (SEM). Normal distribution for all data was analyzed with Shapiro–Wilk-Test. For comparison between two groups, two-tailed Student’s t-test was used for normally distributed data and Mann–Whitney U-test if otherwise. In experiments comparing more than two groups 1-way ANOVA with Tukey or Dunn *post-hoc* tests were implemented. For all tests, p < 0.05 was considered statistically significant. Outliers were detected with the ROUT method. “N” stands for number of animals and “n” designates number of single neutrophils analyzed. The statistical analysis was performed with GraphPad Prism 8 (GraphPad Software, Inc, USA).

## Results

### TNFα priming leads to an increased ROS production in C5a-stimulated neutrophils

**Fig. 1:**
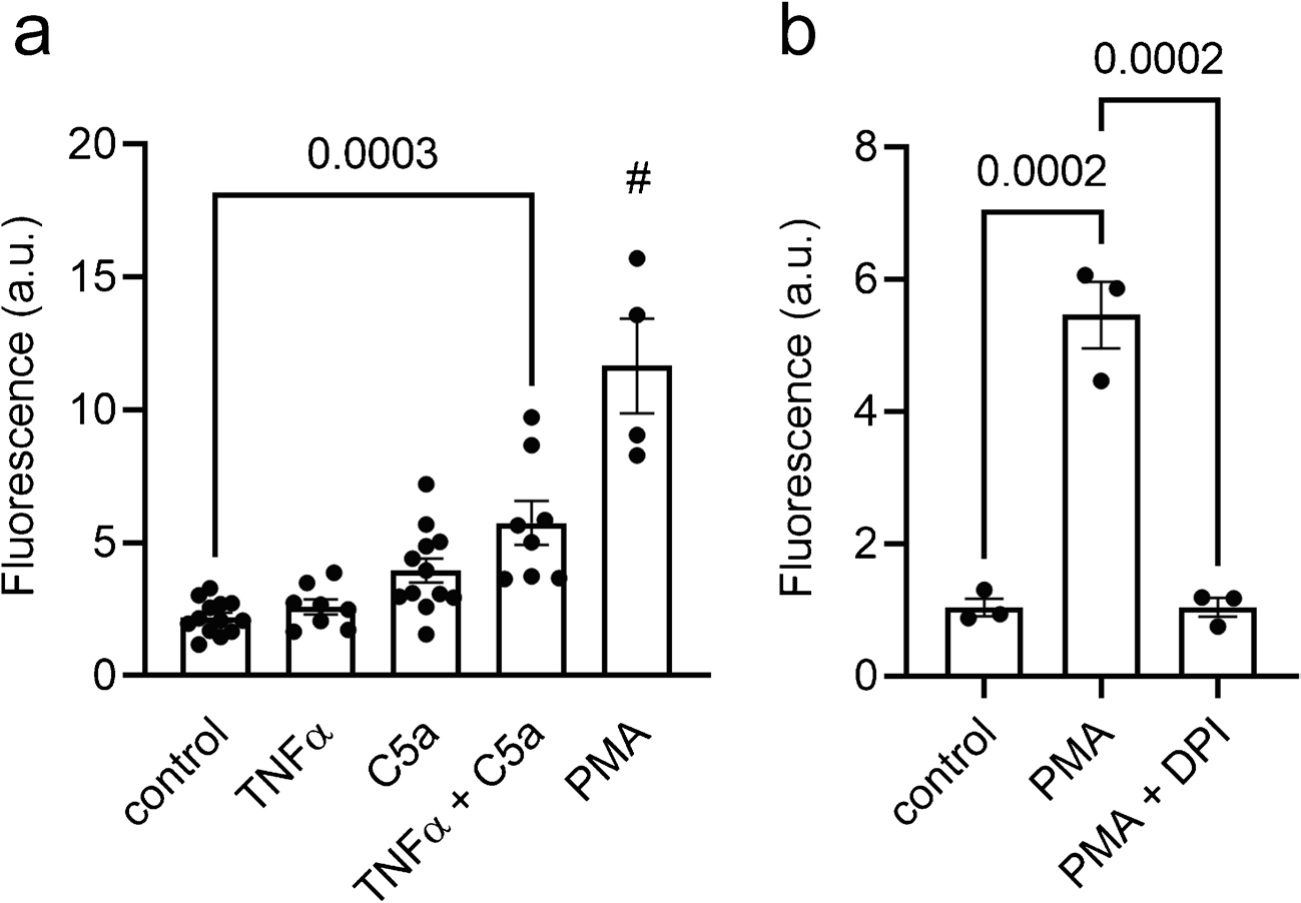
ROS production in murine neutrophils without or with TNFα priming. **a** Intracellular ROS production of murine neutrophils was assessed using the DHR123 oxidation method. Neutrophils were primed with TNFα (20ng/ml = 1.16 nM) for 15 min and afterwards stimulated with C5a (60 nM) or PMA (100 nM) for another 15 min. PMA stimulation served as a positive control. **b** DPI (10 µM) inhibition of the PMA-induced increase in ROS production (N ≧ 8. PMA: *N* = 4. # = p < 0.01 vs. each condition, ANOVA with Tukey post hoc test)

We assessed ROS production of murine neutrophils by measuring the fluorescence of oxidized DHR123. Initially, we tested different concentrations of TNFα for priming neutrophils (5; 10; 20 ng/ml). Stimulation with C5a (60 nM) elicits the highest ROS production in neutrophils that were primed with 20 ng/ml TNFα (data not shown). This concentration was therefore applied in consecutive experiments when indicated. As shown in Fig. 1a, TNFα priming alone does not increase ROS production (TNFα: 2.6 ± 0.3 a.u.; *N* = 8 versus control: 2.2 ± 0.2 a.u.; *N* = 13). However, C5a induces a higher ROS production in TNFα-primed than in unprimed neutrophils (5.8 ± 0.8 a.u.; *N* = 8; versus 4.0 ± 0.5 a.u.; *N* = 12). PMA stimulation served as a positive control since PMA is known as a very strong stimulus for neutrophil ROS production [10]. Indeed, ROS production is highest following stimulation with 100 nM PMA (11.7 ± 1.8 a.u.; *N* = 4.) (Fig. 1a). PMA-induced ROS production is inhibited in the presence of the NOX inhibitor diphenyleneiodonium (DPI; 10 µM; Fig. 1b).

### ROS production inversely correlates with chemokinesis

**Fig. 2:**
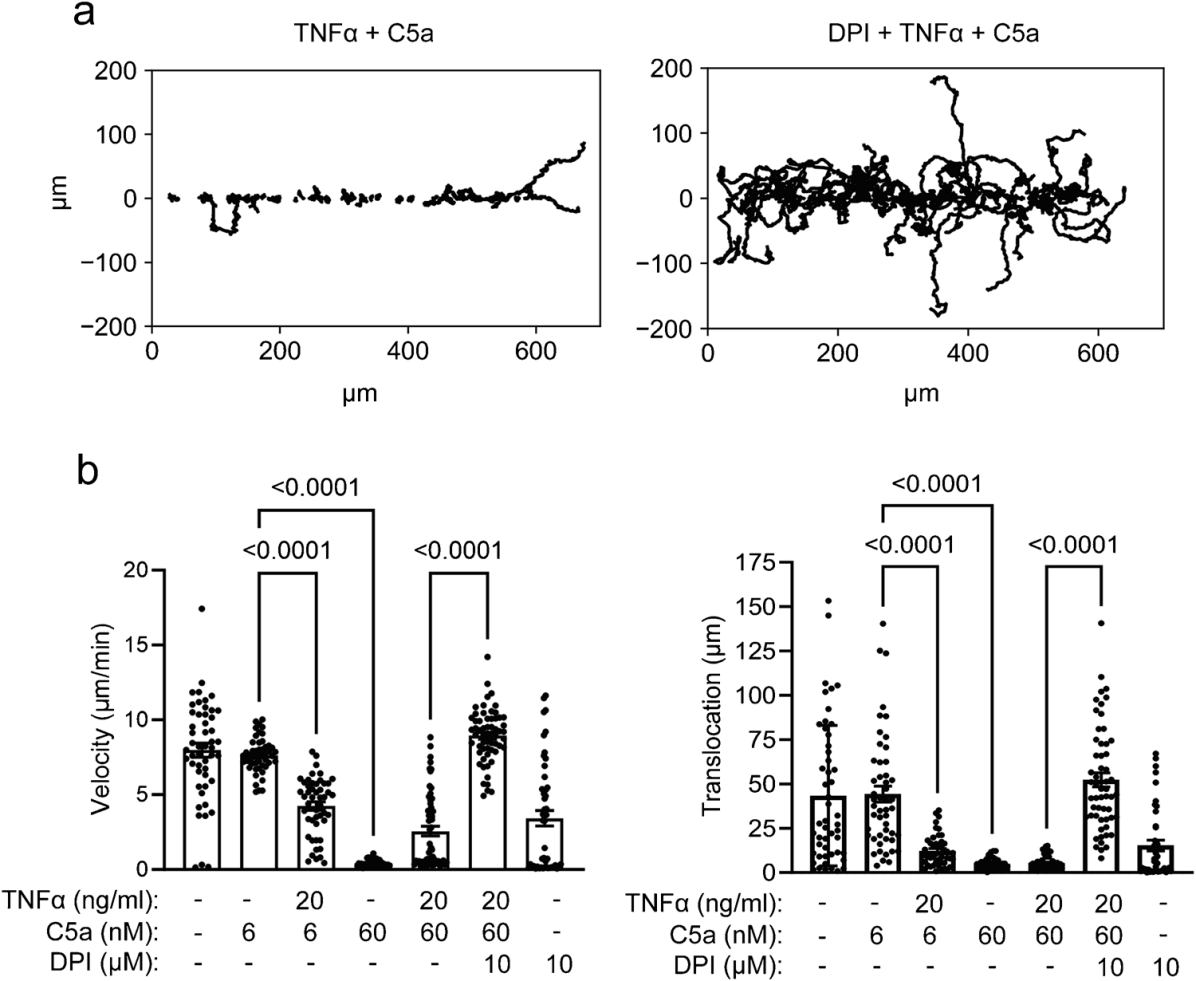
Chemokinetic migration of unstimulated and of C5a-stimulated neutrophils without or with TNFα and/or DPI pretreatment. The neutrophils were primed with TNFα (20 ng/ml) or pretreated with the NOX2 inhibitor DPI (10 µM) when indicated 15 min prior to the stimulation with C5a. **a** Each line corresponds to a trajectory of a single migrating neutrophil. Trajectories are normalized to common starting points along the Y-axis. **b** Velocity and translocation of stimulated neutrophils (*N* = 3, n ≥ 35)

C5a impacts neutrophil chemokinesis in a concentration-dependent manner. The velocity and translocation of neutrophils treated with 6 nM C5a do not differ from those of unstimulated neutrophils of the control group (velocity: 7.6 ± 0.2 µm/min versus 7.9 ± 0.5 µm/min; translocation: 44.3 ± 4.5 µm versus 43.3 ± 5.6 µm/min; N/n = 3/49). TNFα priming slows neutrophils down by ~ 50% when they are treated with 6 nM C5a (velocity: 4.2 ± 0.3 µm/min; translocation: 12.2 ± 1.3 µm; N/n = 3/49). In contrast, stimulating neutrophils with 60 nM C5a leads to a strong adhesion to the fibronectin-coated surface and an almost complete inhibition of chemokinesis (velocity: 0.4 ± 0.05 µm/min; translocation: 4.9 ± 0.5 µm; N/n = 3/35). Under these conditions TNFα priming has no additional effect on neutrophil chemokinesis (velocity: 2.6 ± 0.3 µm/min; translocation: 5.4 ± 0.5 µm; N/n = 3/58) (Fig. 2). Preincubation of TNFα-primed and C5a-stimulated (60 nM) neutrophils with the NOX2 inhibitor DPI (10 µM) rescues chemokinesis (velocity: 8.9 ± 0.2 µm/min; translocation: 52.3 ± 3.9 µm; N/n = 3/60) (Fig. 2). Velocity of DPI-treated neutrophils is similar to that of unstimulated neutrophils (velocity: 3.4 ± 0.5 µm/min; translocation: 15.3 ± 3.0 µm; N/n = 3/49).

### DPI rescues polarized actin polymerization

**Fig. 3:**
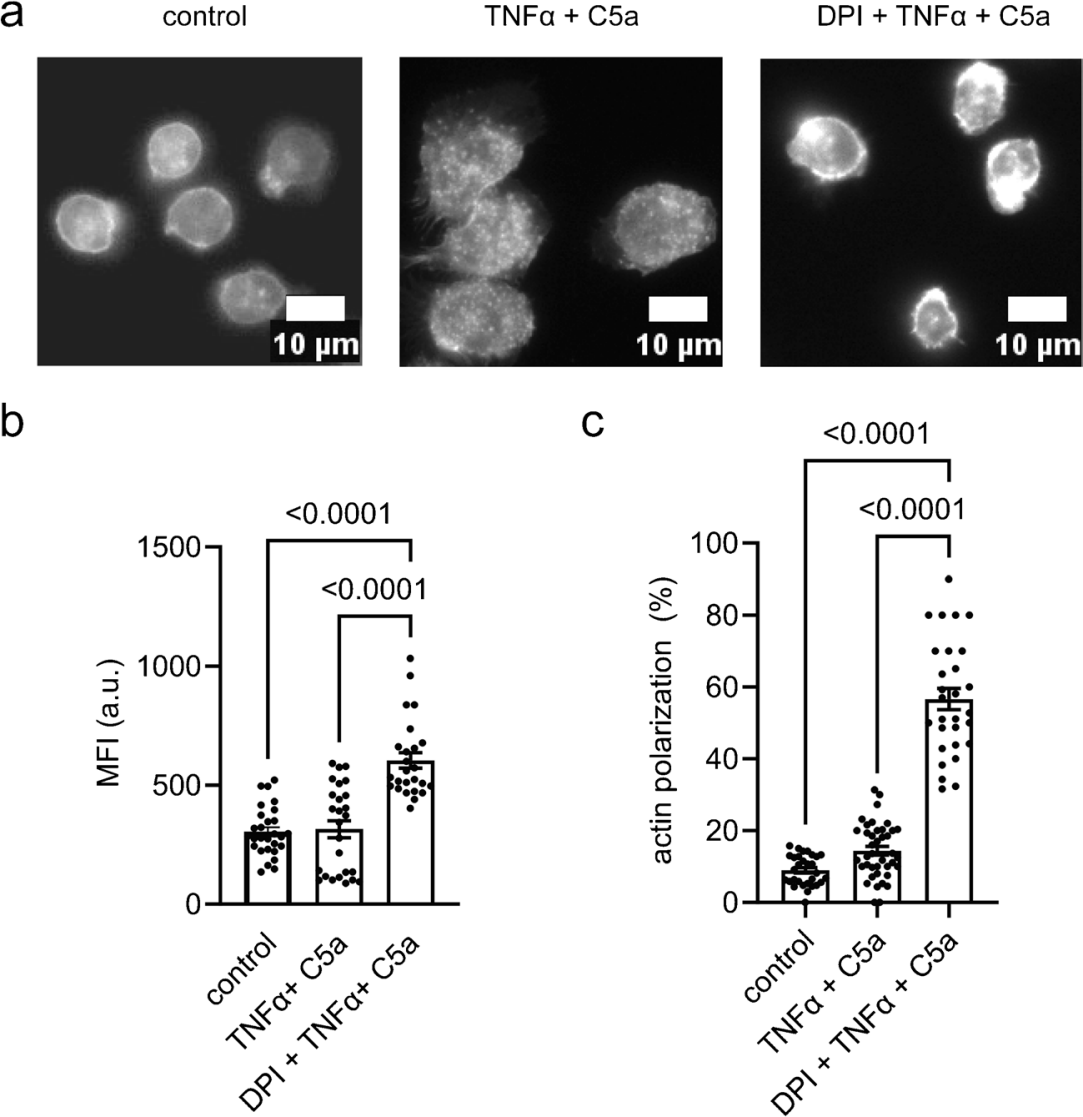
Actin polymerization in primed neutrophils. Prior to the experiment, neutrophils were pretreated with DPI (10 µM), primed with TNFα (20 ng/ml) and stimulated with C5a (60 nM) when indicated. **a** Fluorescence images of TRITC-phalloidin stained neutrophils. Unstimulated, TNFα-primed and C5a-stimulated or DPI-preincubated neutrophils have different actin distribution. **b** Quantification of mean fluorescence intensity (MFI; a.u.) of TRITC-phalloidin stained neutrophils. One dot corresponds to the mean intensity of neutrophils analysed per visual field (N ≧ 3; n ≧ 30 visual fields). **c** Quantification of polarized cells in the visual field (N ≧ 3, n ≧ 30 visual fields)

We analyzed actin polymerization in neutrophils by fluorescence microscopy using TRITC-labeled phalloidin. Unstimulated neutrophils are usually round with a faint ring of polymerized actin underneath the cell membrane. Primed neutrophils stimulated with 60 nM C5a have a characteristic phenotype. They are flattened and appear to be strongly adherent. Their actin cytoskeleton is diffusely distributed with actin punctae at the lower plane of the cell (Fig. 3a). When neutrophils are pretreated with DPI their actin staining pattern changes dramatically. Their actin cytoskeleton forms a ring under the cell membrane and actin polymerization occurs in a polarized way in almost 60% of the cells (Fig. 3a). DPI-treated neutrophils are smaller and not as flattened as primed neutrophils stimulated with 60 nM C5a. Moreover, we rarely observed actin punctae. We used the mean fluorescence intensity as a measure to quantify the DPI-dependent differences in actin polymerization. It amounts to 304.1 ± 19.5 a.u., 315.1 ± 36.0 a.u. and 604.2 ± 32.9 a.u. for unstimulated neutrophils and those stimulated with C5a/TNFα without and with DPI treatment, respectively (Fig. 3b). Under the above-mentioned conditions a polarization of the actin cytoskeleton is evident in 8.9 ± 0.8%, 14.4 ± 1.2% and 56.7 ± 2.9% of the neutrophils, respectively (Fig. 3c).

#### TNFα priming impairs neutrophil chemotaxis in a C5a gradient

**Fig. 4:**
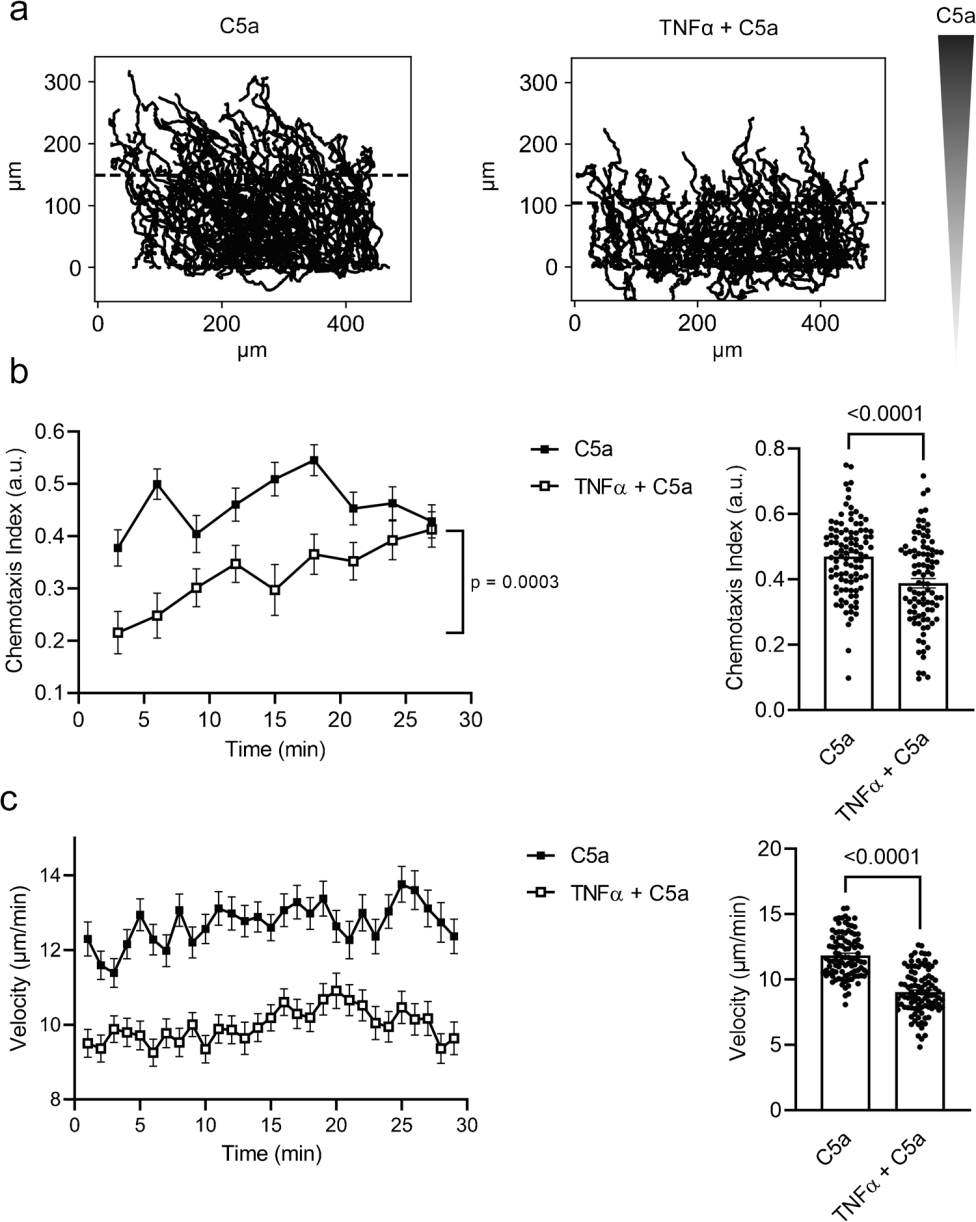
Chemotaxis of neutrophils without or with TNFα priming in a C5a gradient. **a** Each line corresponds to the track of a single neutrophil monitored in a 3D collagen I matrix for 30 min. Neutrophils are unprimed (left) or TNFα-primed (20 ng/ml) (right). The direction of the C5a gradient is indicated on the right-hand side. Trajectories are normalized to common starting points along the y-axis which represents the direction of the C5a-gradient. The dotted horizontal lines indicate the mean distance covered into the direction of the C5a gradient. **b** Chemotaxis index of unprimed and TNFα-primed neutrophils plotted as a function of time (left) (*N* = 5; n ≧ 46. ANOVA with Tukey post hoc test) and mean chemotaxis index (right). **c** Velocity of unprimed and TNFα-primed neutrophils plotted as a function of time (left) and mean velocity of unprimed and TNFα-primed neutrophils (right). (*N* = 5; n ≧ 95. Unpaired t-test)

One of the most important neutrophil functions is to rapidly reach the site of injury or inflammation. We measured the ability to follow chemoattractant gradients by exposing neutrophils to a C5a gradient. To mimic their physiological environment in a tissue, they were embedded in a 3D collagen I matrix. We compared unprimed and TNFα-primed (20 ng/ml) neutrophils (see Online Resources 1 & 2). C5a and TNFα were used in concentrations that lead to the highest ROS production in our experiments (see above, Fig. 1). The trajectories of unprimed and TNFα-primed neutrophils are shown in Fig. 4a. The mean distance covered within the gradient is indicated by the dotted horizontal lines. To quantify chemotaxis, we analyzed chemotaxis index and velocity. TNFα priming leads to a significant reduction of the chemotaxis index by ~ 20% compared to unprimed neutrophils when calculating the mean value for the entire course of the experiment (0.39 ± 0.01 a.u.; N/n = 5/95 versus 0.47 ± 0.01 a.u.; N/n = 5/100) (Fig. 4b). When plotting the chemotaxis index as a function of time it becomes evident that chemotaxis of primed neutrophils becomes more efficient towards the end of the experiment (CI = 0.41 ± 0.03 a.u.; N/n = 5/100) than at its start (CI = 0.22 ± 0.04 a.u.; N/n = 5/100). Stated differently, chemotaxis of primed neutrophils improves when they are moving into areas with higher C5a concentration towards the end of the experiment. Accordingly, the chemotaxis index of primed neutrophils with starting points in the upper half of the visual field is higher (CI = 0.43 ± 0.02 a.u.; N/n = 5/46) than the chemotaxis index of primed neutrophils starting in the lower half of the visual field (CI = 0.35 ± 0.02 a.u.; N/n = 5/49) (data not shown). Unprimed neutrophils chemotax with constant efficiency during the entire course of the experiment (Fig. 4b). Velocity is also reduced upon TNFα priming. Priming causes a reduction of velocity by ~ 25% (11.9 ± 0.2 µm/min; N/n = 5/99 versus 9.0 ± 0.2 µm/min; N/n = 5/100) (Fig. 4c). Thus, neutrophils follow the C5a gradient faster and in a more directed way when they are not primed with TNFα.

### Neutrophil stimulation with chemoattractants causes membrane depolarization

**Fig. 5:**
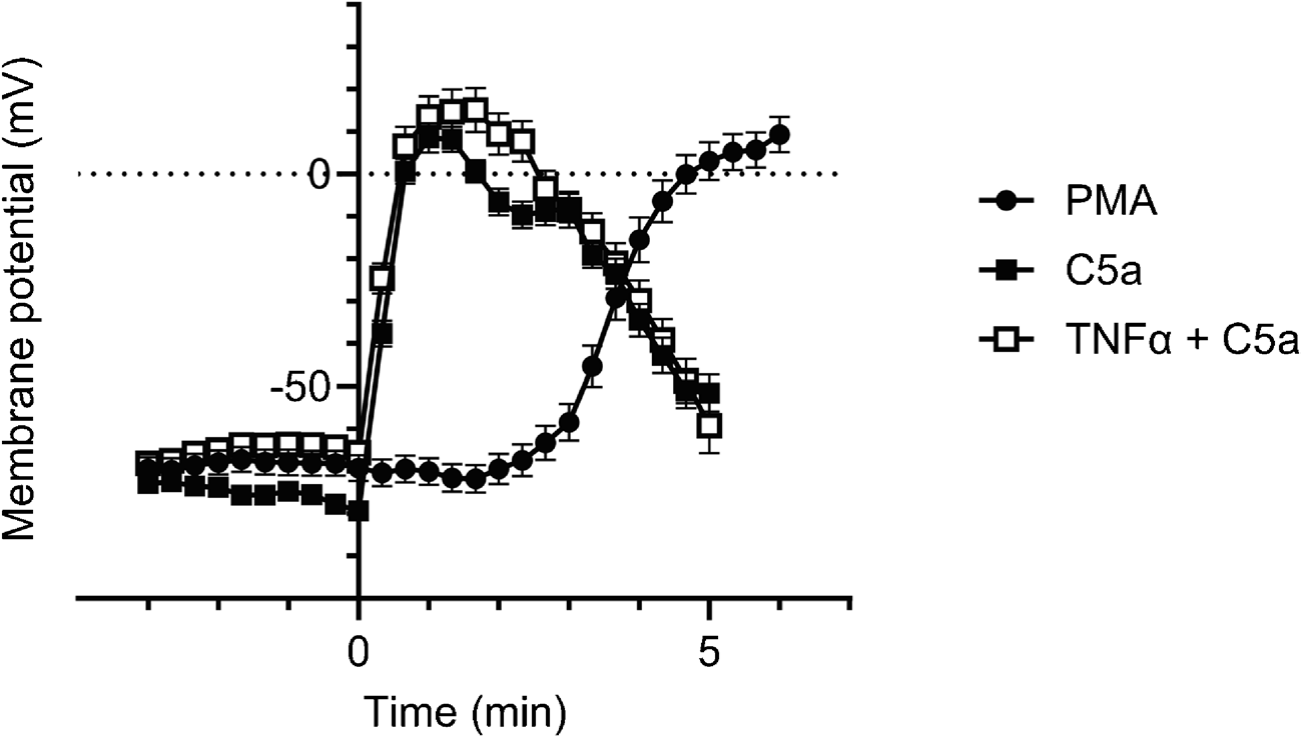
Membrane potential of neutrophils plotted as a function of time. The membrane potential was measured with the voltage-sensitive fluorescent dye DiBaC_4_(3) (2 µM). Neutrophils were unprimed or TNFα-primed (20 ng/ml) before the experiment and stimulated with C5a (60 nM) or PMA (100 nM) at t = 0 min. (N ≧ 4; n ≧ 58)

The knowledge about membrane potential dynamics and its role in neutrophil function is at best only rudimentary. Here, we performed membrane potential measurements in order to explore its dynamics under conditions of increased ROS production with physiological stimuli. We used live-cell imaging with the voltage-sensitive fluorescent dye DiBaC_4_(3) and determined the initial response within the first 5 – 10 min of stimulation. Neutrophils were unprimed or TNFα-primed (20 ng/ml) before the experiment and stimulated with C5a (60 nM) or PMA (100 nM) at t = 0 min. The resting membrane potential amounts to -74.3 ± 0.7 mV (N/n = 6/93) for unprimed neutrophils and -67.2 ± 1.2 mV (N/n = 3/58) for TNFα-primed neutrophils. PMA and C5a stimulation both lead to a massive depolarization of the membrane potential by ~ 80 mV (from ~ -70 mV to ~  + 10mV) (Fig. 5). However, the time course of the depolarization varies. Stimulation of unprimed neutrophils with 60 nM C5a causes an almost instantaneous depolarization by 86.6 ± 4.2 mV within the first min. The peak value of + 8.6 ± 3.4 mV is reached after 1 min (N/n = 3/72). The corresponding values for TNFα-primed neutrophils are a depolarization by 79.1 ± 4,5 mV within the first min and a peak value of 15.1 ± 5.2 mV after 1.6 min (N/n = 3/58). This rapid initial depolarization (86.6 mV/min and 79.1 mV/min for unprimed and primed neutrophils, respectively) is transient. It is followed by a slower repolarization (16.1 mV/min and 21.9 mV/min for unprimed and primed neutrophils, respectively). In contrast, PMA stimulation leads to a delayed but persistent depolarization. It reaches a value of 9.3 ± 4.1 mV at t = 6 min (N/n = 9/114) (Fig. 5).

So far, the functional relevance of these enormous membrane potential dynamics for neutrophil migration is unknown.

### Initial depolarization of the membrane potential is accompanied by a decreased migration velocity

We wanted to investigate whether there is a link between membrane potential dynamics and neutrophil migration or ROS production. We therefore applied a multi-parametric analysis of the measurements of the cell membrane potential allowing the simultaneous determination of membrane potential and migration. Thus, we could directly link membrane potential values of neutrophils shown in Fig. 5 to their respective migratory behavior.

**Fig. 6:**
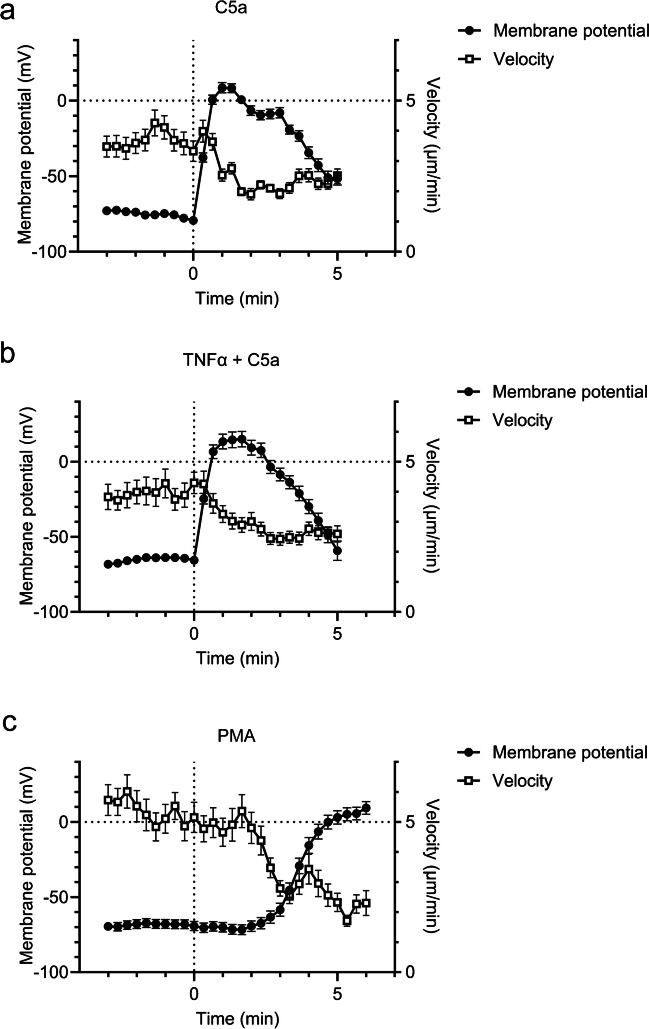
Membrane potential and velocity of neutrophils following stimulation with C5a or PMA. When indicated, neutrophils were primed with TNFα (20 ng/ml) 15 min prior to experiment. C5a and PMA were added at t = 0. Dynamic changes of membrane potential and velocity of **a** unprimed neutrophils stimulated with C5a (60 nM), **b** TNFα-primed neutrophils stimulated with 60 nM C5a and **c** unprimed neutrophils stimulated with PMA (100 nM). (N/n ≧ 4/58)

Under the conditions of our assay, migration velocity of unstimulated neutrophils amounts to 3.7 ± 0.1 µm/min for unprimed (N/n = 6/93) and 3.9 ± 0.1 µm/min for primed neutrophils (N/n = 3/58). The rapid depolarization of the membrane potential by 86.6 ± 4.2 mV within the first min upon C5a stimulation (60 nM) is accompanied by a gradually decreasing velocity to 1.9 ± 0.2 µm/min within 3 min. The decrease of the velocity is concurrent with the depolarization (Fig. 6a; N/n = 3/72). In TNFα-primed neutrophils the membrane potential initially depolarizes by 79.1 ± 4.5 mV and the velocity drops to 2.4 ± 0.2 µm/min at t = 3 min (Fig. 6b; N/n = 3/58). The curves are similar for unprimed and primed neutrophils with C5a stimulation (60 nM). After PMA (100 nM) stimulation, the delayed depolarization by 78.5 ± 4.6 mV within 6 min is accompanied by a decrease of the velocity from 5.1 ± 0.5 µm/min at t = 0 to 2.3 ± 0.4 µm/min at t = 6 min (Fig. 6c; N/n = 9/114).

## Discussion

Neutrophils are circulating in the blood in a resting state. Upon infection, pro-inflammatory mediators such as TNFα or granulocyte-colony stimulating factor (G-CSF) are released by various cells at the inflammatory site, primarily by tissue macrophages. These mediators cause neutrophil priming which ‘boosts’ the activation induced by a secondary stimulus [13]. They also lead to a strong activation of endothelial cells thereby triggering neutrophil adhesion and recruitment [27, 36, 40]. After crossing the endothelial barrier, neutrophils migrate towards the infection site along a gradient of chemoattractants such as C5a [27]. C5a that is released upon inflammation can also damage the glycocalyx of endothelial cells [43]. Thus, the first contact of neutrophils with C5a occurs intravascularly. The aim of our study was to monitor the initial response of unprimed and primed neutrophils following C5a stimulation. We were particularly interested in membrane potential dynamics. How is the membrane potential affected by neutrophil stimulation and does it play a role in coordinating motility and ROS production?

It is well established that ROS production leads to depolarization of the membrane potential. This is caused by the activation of the NADPH oxidase (NOX2) [6, 22] that mediates an outwardly directed electron current [8, 39]. The depolarization of the cell membrane potential is at least partially compensated by voltage-gated proton channels (H_V_1) [9, 18, 37]. Many of these studies used PMA as a stimulus for ROS production in suspended neutrophils. In these experiments the onset of the depolarization was usually rather slow and required several minutes. PMA activates NOX2 in a way that is independent of the signaling cascades triggered by the stimulation of chemoattractant receptors [3, 5, 14]. The onset of PMA-induced ROS production is typically delayed by 2 – 4 min [12]. In our hands, it takes more than 3 min upon PMA stimulation of adherent neutrophils until a depolarization of ~ 20 mV is reached. The protracted time course of both, PMA-induced ROS production and membrane potential dynamics, is consistent with NOX2 activity causing the membrane depolarization.

In contrast, stimulation of adherent and migrating neutrophils with C5a causes a very rapid and massive depolarization of the membrane potential. It rises by ~ 40 mV within the first 20 s and by 80—90 mV in less than one minute. PMA elicits a similar depolarization only after ~ 6 min. It is noteworthy that the amplitude of the depolarization is comparable to that of action potentials of excitable cells such as neurons. The simultaneous measurement of membrane potential dynamics and neutrophil migration revealed that the initial depolarization is paralleled by a reduction of the neutrophil velocity. These observations raise the following three questions: What causes the initial depolarization after C5a stimulation, why is it transient and why does it impede neutrophil motility?

Several lines of evidence suggest that the C5a-induced depolarization of the membrane potential is not due to NOX2-mediated electron transport. First, it is much faster and more transient than the depolarization caused by PMA. In endothelial cells C5a-stimulated and NOX-dependent ROS production occurred only after a lag period of approximately 5 min [42]. In contrast to ROS production which we only observed in primed neutrophils, the rapid initial depolarization occurs independently of whether neutrophils have been primed or not. Unprimed neutrophils stimulated with 60 nM C5a depolarize essentially as rapidly as primed ones. Finally, PMA stimulation leads to much more ROS production than C5a, but the maximum depolarization of the membrane potential is nearly the same for PMA and C5a.

Our experiments do not disclose the mechanisms underlying the C5a-induced, transient depolarization of the membrane potential. However, based on earlier reports it is likely that C5a triggers G-protein coupled BLT1 receptors which then initiate a fast and depolarizing activation of Ca^2+^ influx via ORAI channels [9, 16, 41]. C5a triggers a transient increase of the intracellular Ca^2+^ concentration with a similar time course as the transient depolarization of the membrane potential observed in our study [41].

For repolarization cation efflux or anion influx is needed. H^+^ efflux via voltage-gated proton channels (H_V_1) cannot be ruled out. In addition, neutrophils express at least three different types of K^+^ channels—K_V_1.3 [20], K_ir_2.1 [30] and K_Ca_3.1 [16, 19]—whose temporally fine-tuned activities could cause the repolarization. Finally, also NCX1.3 contributes to the initial repolarization. Since the intra- and extracellular Na^+^ and Ca^2+^ concentrations as well as the membrane potential of neutrophils are known from our [32] and other groups' previous works [41] as well as from this study its reversal potential can be calculated. The calculation shows that NCX1.3 operates in the reverse mode – import of 1 Ca^2+^ ion in exchange for the export of 3 Na^+^ ions – during the peak depolarization ([Na^+^]_i_ = 17 mmol/l, [Ca^2+^]_i_ = 0.0004 mmol/l, [Na^+^]_e_ = 140 mmol/l, [Ca^2+^]_e_ = 1.2 mmol/l and V_M_ = 0.015 V) [1, 2, 29, 32]. Thereby it also transiently contributes to the repolarization and the increase of the intracellular Ca^2+^ concentration. In contrast, under resting conditions ([Na^+^]_i_ = 12 mmol/l, [Ca^2+^]_i_ = 0.0001 mmol/l and V_M_ = -0.07 V) NCX1.3 is operating in the forward mode. In our view this is an intriguing parallel to cardiomyocytes in which NCX switches its transport direction during the action potential [2]. Clearly, more studies are needed to unravel the molecular mechanisms underlying membrane potential dynamics of neutrophils.

The question remains whether cell membrane potential dynamics impact on the migratory behavior of neutrophils. At first sight there appears to be a correlation on a short time scale. Migration speed is reduced during the initial depolarization of the cell membrane potential. This is particularly evident in PMA-treated neutrophils [4]. However, the repolarization of the membrane potential appears not to be sufficient to "rescue" the migratory speed. Unprimed and primed neutrophils exhibit very similar initial membrane potential dynamics after C5a-stimulation. Yet, their migratory behavior differs when observed for a longer time period. Importantly, primed neutrophils produce ROS when stimulated with C5a which is not the case for unprimed cells. ROS in turn is known to inhibit Orai1 channels [33]. One could speculate that the resulting reduction of Ca^2+^ influx contributes to reduced motility. NOX2-generated ROS is also known to reduce actin polymerization through actin glutathionylation and thereby impair neutrophil chemotaxis ([38]; Fig. 3). Consistent with these observations we found that the NOX2-inhibitor DPI rescues chemokinesis in TNFα-primed and C5a-stimulated neutrophils (Fig. 4). We are aware of the fact that DPI also blocks other flavin-containing enzymes involved in mitochondrial respiration by inhibiting FMN- and FAD-dependent enzymes of Complex I and II. Thereby it reduces mitochondrial ATP production and oxidative mitochondrial metabolism [35]. However, this effect of DPI is rather unlikely to play a major role in our experiments. Efficient neutrophil chemotaxis requires not only a polarized actin cytoskeleton, but also energy supplied by mitochondrial metabolism [31]. Inhibition of mitochondrial ATP production is not compatible with our observations of rescued polarization and chemokinesis by DPI in TNFα-primed and C5a-stimulated neutrophils. Finally, we cannot rule out that other mechanisms also contribute to reduced migration when neutrophils produce ROS.

In summary, our study is one of the first simultaneously measuring membrane potential dynamics and migration of neutrophils by means of live-cell imaging. Our results revealed a temporal relation between membrane potential dynamics and migration of neutrophils that is dependent on the type of stimulus. Thus, different membrane potential dynamics can be assigned to different means of stimulation. It remains to be determined in future studies whether membrane potential dynamics are the outcome of upstream signaling events or whether they have additional signaling properties themselves.

### Supplementary Information

Below is the link to the electronic supplementary material.

Supplementary file1 **Online Resource 1 **Murine neutrophils migrate towards a C5a gradient in a 3D collagen-I-matrix. After 10 minutes of incubation neutrophils were recorded for 30 minutes. We used Zeiss Axiovert 40 C and Axiovert 25 microscopes and acquired the images with Bresser MikroCam SP 3.1 cameras. Images were taken every 5 s using MikroCamLab II software. Use scale for size reference. (AVI 18995 KB)

Supplementary file2 **Online Resource 2 **TNFα-primed neutrophils migrate towards a C5a gradient in a 3D collagen-I-matrix. After 10 minutes incubation neutrophils were recorded for 30 minutes. We used Zeiss Axiovert 40 C and Axiovert 25 microscopes and acquired the images with Bresser MikroCam SP 3.1 cameras. Images were taken every 5 s using MikroCamLab II software. Use scale for size reference. (AVI 20897 KB)

## Data Availability

Not applicable.
